# DNA Barcoding for Tracing Biodiversity in Mixed Crop Food Products: A Proof of Concept Within the BioValue Project

**DOI:** 10.3390/foods14183256

**Published:** 2025-09-19

**Authors:** Maria-Dimitra Tsolakidou, Nikolaos Nikoloudakis, Cyril Tisseyre, Marija Knez, Eleonora Barilli, Konstadinos Mattas, Andreas Katsiotis

**Affiliations:** 1 Department of Agricultural Sciences, Biotechnology and Food Science, Cyprus University of Technology, 3603 Limassol, Cyprus; maria.tsolakidou@cut.ac.cy; 2 ECOZEPT France SAS, Le Barcelone, Bât. 12, 145 Rue Guillaume Janvier, FR-34070 Montpellier, France; tisseyre@ecozept.com; 3 Capacity Development in Nutrition, 11000 Belgrade, Serbia; marijaknez186@gmail.com; 4 Center of Research Excellence in Nutrition and Metabolism, Group for Nutrition and Metabolism, Institute for Medical Research, National Institute of Republic of Serbia, University of Belgrade, 11000 Belgrade, Serbia; 5 Solintagro SL, Calle Escritora Rosa Chacel 4 Local 2, 14004 Cordoba, Spain; e.nora@libero.it; 6 Department of Agricultural Economics, Aristotle University of Thessaloniki, 54124 Thessaloniki, Greece; mattas@auth.gr

**Keywords:** biodiversity, DNA barcoding, food authenticity, food products, plant species

## Abstract

In a world of rapidly globalizing food markets, biodiversity, authenticity, and the safety of food products have become a universal concern. DNA barcoding is a widely used molecular-based method that can identify biological material and is used for the traceability of both raw materials and ingredients in processed food. In the present study, contacted within the framework of the BioValue Horizon Project, which promotes the role of agrobiodiversity in sustainable food systems, DNA barcoding using the *ITS* and *rbcL* markers was employed as a proof-of-concept approach to reveal the biodiversity and authenticity of ten commercial plant-based products. Following successful DNA amplification and sequencing using six products as a proof-of-concept, a diverse range of plant genera and species were identified, verifying biodiversity. A strong correlation between *ITS* and *rbcL*-based markers was demonstrated, supporting their combined use for reliable species-level biodiversity assessment. Finally, heat map analysis of label contents and sequencing-based genera identification confirmed high concordance between label claims and sequencing results in most cases, though undeclared species and absent labeled taxa were also detected, highlighting potential mislabeling or cross-contamination.

## 1. Introduction

The food supply chain is widely recognized as a multidisciplinary and complex sector that integrates various elements across multiple levels and stakeholders, and its sustainability and biodiversity are central themes of current research initiatives such as the BioValue Horizon Project (https://www.biovalue-project.eu/, accessed on 17 September 2025). Throughout the different stages of production, there are critical checkpoints that are vital for ensuring product quality. Farm (and Fork) biodiversity in particular is increasingly seen as a key indicator of eco-friendly farming practices, significantly affecting the final product. Biodiversity is the full spectrum of taxa diversity at genetic, species, and ecosystem levels that sustains and enriches food production systems and diets, making it foundational to global food security, nutrition, sustainability, and ecosystem health [1,2]. Food biodiversity includes domesticated plants and animals, wild relatives of domesticated species, wild species harvested for food and other products, and a vast range of organisms that live in and around food and agricultural production systems, sustaining them and contributing to their output [1].

Over the past few years, it has been clearly demonstrated that biodiversity is essential for sustaining food systems, ensuring that they provide nutritious food with minimal negative impacts on the environment, and promoting human well-being [3,4]. However, the genetic diversity in crops and animals, which ensures the production of sufficient and nutritious food and the resilience of agroecosystems against different environmental stresses, is rapidly declining due to climate change, monoculture (as the outcome of green revolution), and the uncontrollable agricultural expansion [5,6,7]. Biodiversity loss could undermine the resilience of food systems and the stability of food production, leading to negative effects for food security [8].

The ongoing global pressure to meet rising food demand has contributed to biodiversity loss worldwide, which has been accelerating in recent decades due to the reliance on conventional food systems [9,10]. This creates a paradox scheme in which biodiversity is essential for sustaining food systems, yet at the same time, conventional food systems are one of the most important threats to biodiversity [9]. According to FAO, 75% of plant genetic diversity has been lost and two-thirds of global plant-derived food is provided by only three major crops—maize (*Zea mays*), wheat (*Triticum aestivum*), and rice (*Oryza sativa*) [1,11]. Moreover, the constantly increasing demand and consumption of processed and ultra-processed food products manufactured with ingredients derived from these few high-yielding plant crops has a negative impact on biodiversity, and their contribution to plant biodiversity is often unclear or underreported [12,13].

Given these circumstances, food labeling may not always reflect the true botanical composition of a product, particularly when ingredients are processed into forms that obscure morphological identification [14,15]. To ensure transparency and traceability for customers, regulators, and researchers aiming to promote plant and food biodiversity, reliable identification systems are necessary to guarantee adequate authenticity checkpoints throughout the entire supply chain and distribution networks. Accurate identification of genera, species, and the varieties used is essential not only to retrieve information on their origin but also to authenticate the raw materials and related processed products distributed both at the local and global scale [16].

DNA barcoding is a molecular scheme that has been used in recent years to identify wild and cultivated plant species and has served as an indicator of biological diversity [17,18]. This method relies on identifying species using specific regions of their DNA and over the last decade has emerged as a key tool in food authentication, mislabeling, food fraud, allergens detection, and biodiversity assessment [14,16,19,20]. Although DNA barcoding involves laboratory procedures and expert analysis, the use of open-access databases and decreasing sequencing costs make this approach increasingly feasible for the food industry, particularly for verifying authenticity and traceability of high-value or complex products. DNA barcoding is particularly useful for identifying plant species in food products, especially when the physical or morphological characteristics of the ingredients are altered during processing, allowing the detection and verification of the plant species used, even in complex or highly processed products [21]. In plants, among the most commonly used regions for barcoding are the chloroplastic *ribulose-bisphosphate carboxylase* (*RuBisCo*) *enzyme* (*rbcL*), and the nuclear *internal transcribed spacer* (*ITS*), as their combination offers precise species-level identification [15]. *rbcL* gene is highly conserved across plant species, making it ideal for broad taxonomic identification whereas *ITS* genes offer high variability enabling species identification [22,23].

In this context, the scope of the present study was to develop a proof-of-concept scheme, using DNA barcoding, for identifying different taxa within plant-based food products and assess the contribution of biodiversity in the biological value (BioValue) of these products. The term BioValue in this work comes from the acronym of the BioValue Horizon project (https://www.biovalue-project.eu/, accessed on 17 September 2025). BioValue is a research initiative that aims to emphasize the role of agrobiodiversity in strengthening sustainable food systems, the agro-food value chain, the environment, and consumer preferences and health. Within this framework, besides composition reflecting food biodiversity, products were also selected based on the degree of processing to assess the combined effect on DNA-based identification and traceability. A range of products, including legumes, seeds, vegetable mixes, pasta, and tomato-based products, were selected, as they represent both biodiversity-rich foods and different processing levels (dried, frozen, canned, and thermally processed), thereby ensuring that the study evaluated DNA barcoding performance under realistic market conditions. The main DNA extraction methods were evaluated, and *ITS*/*rbcL* gene regions were amplified across six selected plant-based food products. Amplicons were sequenced and compared against databases to assess the degree of biodiversity in each product.

## 2. Materials and Methods

### 2.1. Plant-Based Food Products

For the assessment of biodiversity, a comprehensive screening was conducted on a wide assortment of plant-based commercial food products (aligned with BIOVALUE) with diverse ingredient composition and processing methods. The selected commodities that varied in salt and sugar contents, additives, and thermal processing techniques are shown in Table 1. All products were purchased from commercial food markets. Dried legumes, seed mixtures, and pasta products were homogenized with a grinder and stored at −20 °C, while the frozen blend and the canned mix of legumes and vegetables were homogenized with a mortar and pestle in the presence of liquid nitrogen and then stored in a deep freezer. Tomato-based products were also homogenized using a mortar and pestle and stored at −20 °C. In all cases, the entire package content was used to ensure representativeness. From each homogenized sample, 10–30 mg of dried products and100–200 mg of frozen, canned, or raw products were used for DNA extraction.

### 2.2. DNA Extraction

To identify the most effective DNA extraction method, three techniques were employed, considering that thermal processing and additives can impact DNA integrity and isolation. Two commercial silica column-based kits, as well as a CTAB-based protocol [24], were evaluated. To mitigate the interference of phenolic compounds, which can inhibit DNA isolation, all samples were pre-washed with Sorbitol Washing Buffer twice before extraction as previously reported [25]. For the two commercial kits used, the manufacturers’ protocols were followed. The CTAB-based method was performed with slight modifications. One hundred mg of tissue per sample was homogenized with 1 mL of CTAB buffer and incubated at 65 °C for 20 min with agitation at 600 rpm in a thermo-mixer (HCM100-PRO, DLAB Scientific Co., Ltd., Beijing, China). Subsequently, 5 µL of RNase (10 mg/mL) was added, and the mixture was incubated at room temperature for 15 min to remove RNA traces. Then, 700 µL of phenol-chloroform-isoamyl alcohol (25:24:1) was added, and the solution was vortexed vigorously. The mixture was centrifuged at 10,000 rpm for 15 min at 4 °C, and the upper aqueous phase was collected. To further purify the DNA, an additional step was introduced. Half volume of 5 M NaCl was added to the sample, mixed gently by inversion, followed by the addition of 3 volumes of cold 95% ethanol [26]. The mixture was then placed in a −20 °C freezer and incubated for 1 h to precipitate the DNA. After incubation and centrifugation (15 min at 4 °C), the liquid was discarded, and the DNA pellet was washed with 70% ethanol. Finally, the dried pellet was dissolved in 20–30 µL of deionized water (ddH_2_O). The quality and quantity of the extracted DNA samples were evaluated using a Nano Drop 2000 spectrophotometer (Thermo Scientific, Waltham, MA, USA).

### 2.3. PCR Amplification

To identify the presence of the different taxa within the tested products, PCR amplification was employed to construct DNA libraries. For accurate sequencing results, amplification targeted two distinct DNA regions: the Internal Transcribed Spacers (*ITS1* and *ITS2*) of the *ribosomal DNA* (*rDNA*) gene, and the chloroplastic gene *rbcL*, which facilitates the refinement of phylogenies among seed plants. For both amplified regions, universal interspecies primers were used (forward primer 18S: 5′-ACG-AAT-TCA-TGG-TCC-GGT-GAA-GTG-TTC-G-3′, reverse primer 26S: 5′-TAG-AAT-TCC-CCG-GTT-CGC-TCG-CCG-TTA-C-3′, rbcL-1F: 5′-TG-TCA-CCA-CAA-ACA-GAA-AC-3′, and rbcL-724R: 5′-TCG-CAT-GTA-CCT-GCA-GTA-GC-3′) as previously reported [27,28,29,30,31,32]. PCR amplification was carried out using KAPA DNA polymerase (KAPA Biosystems) with 20 ng of template DNA per reaction. Reaction mixtures were prepared following the manufacturer’s instructions, with a final primer concentration of 0.8 μM. The PCR conditions were set as follows: an initial denaturation at 94 °C for 5 min was performed to ensure complete disassociation of DNA. This was followed by 44 cycles of denaturation at 94 °C for 40 s, annealing at 55 °C for 40 s, and extension at 72 °C for 40 s. A final extension step was conducted at 72 °C for 60 s to complete the amplification process.

### 2.4. Long-Read Nanopore Sequencing of ITS and rbcL rRNA Regions

Next Generation Sequencing (NGS) was performed using a standard protocol as provided by BioISI Genomics (Lisbon, Portugal). Amplification products were purified using the Solid Phase Reversible Immobilization (SPRI) technique with magnetic beads [33]. PCR product purity was assessed by absorbance at 260 nm using a NanoDrop 1000 Spectrophotometer (Thermo Fischer Scientific, Waltham, MA, USA), and the concentration by Qubit™ 4 Fluorometer (Thermo Fischer Scientific, Waltham, MA, USA). The library was prepared from 200 fmol input DNA from each sample using the Sequencing Native Barcoding Kit V14 (SQK-NBD114.96) (Oxford Nanopore Technologies, Oxford, UK) in accordance with the manufacturer’s protocol. Sequencing runs were performed using R10.4.1 flow cells on a PromethION sequencing platform, and sequencing data were acquired in real time using MinKNOW 24.02.10 software.

### 2.5. Bioinformatic Analysis

Sequencing data were derived from *ITS* and *rbcL* amplicons, with the exclusion of low-quality reads. The remaining reads underwent size selection, retaining those with lengths between 800 bps and 1200 bps, accomplished through prinseq-lite [34]. Taxonomic classification employed a Lowest Common Ancestor approach, utilizing an index based on k-mers that map to the lowest common ancestor of all genomes known to encompass a specific k-mer [35]. Genera and species plots were generated by microbiome R package (version 1.30.0).

### 2.6. Statistical Analysis

Statistical analysis was performed using IBM SPSS Statistics 25 software. *ITS* and *rbcL*-based sequencing data were subjected to Pearson correlation analysis. The Pearson correlation coefficient (*r*) and associated *p*-value were calculated to assess the strength and statistical significance of the correlation.

## 3. Results and Discussion

### 3.1. DNA Extraction and PCR Performance Are Affected by Food Processing and Additives

The products included in the present study were selected to reflect a broad range of plant-based ingredients, promoting biodiversity, and different processing levels. Different DNA extraction methods were performed to determine the most effective protocol tailor-made for various commodities. Among the methods tested, the modified CTAB-based protocol provided the highest quality and higher levels of DNA quantity, particularly for products with higher thermal processing and complex additive composition as proven previously [36,37,38,39]. This was further confirmed by Nanodrop readings, where the CTAB method consistently yielded better A260/A280 and A260/A230 ratios, indicating higher purity and concentration of the extracted DNA (Supplementary Table S1). In particular, all ten products demonstrated higher DNA yields, and the 260/230 ratio was profoundly higher compared to the results obtained using silica column-based DNA extraction, revealed by Nanodrop. However, it was evident that products subjected to excessive thermal processing (e.g., Product 5: lentil pasta) or contained additives (e.g., Products 7, 8, 10: canned and dried tomatoes) still presented challenges for DNA extraction even with the CTAB method, which had otherwise produced better results.

Based on these results, it was demonstrated that products with high concentrations of phenolic compounds, salts, sugars, and additives such as canned tomatoes, legumes, and vegetables along with those subjected to excessive thermal processing, like pasta or pre-cooked meals, presented significant restrictions for both DNA isolation and PCR amplification (Supplementary Figure S1) [40]. These components act as potential inhibitors, complicating the extraction and amplification processes. This is one of the major limitations of DNA barcoding, as high-heat cooking, chemical treatments, or long-term storage results in DNA degradation and reduces the ability of DNA barcoding to recover intact sequences for analysis [41]. According to Lorusso and colleagues, this limitation means that DNA barcoding may not always provide accurate results for highly processed foods, when the DNA quality is compromised [42]. As a result, products 1–6 (Table 1) that demonstrated high DNA quality and successful PCR amplification of both the *ITS* and *rbcL* genes were selected for further sequencing analysis. The six selected products contained legumes, vegetable and seed mixtures and processed products (lentil, wheat pasta, and ready frozen meals). No canned products were included as they did not give any DNA of acceptable integrity, or PCR amplification was inhibited.

### 3.2. DNA Barcoding Using ITS and rbcL Genes Reveals Consistently Diverse Taxonomic Composition in Commercial Plant-Based Products

**Figure 1:**
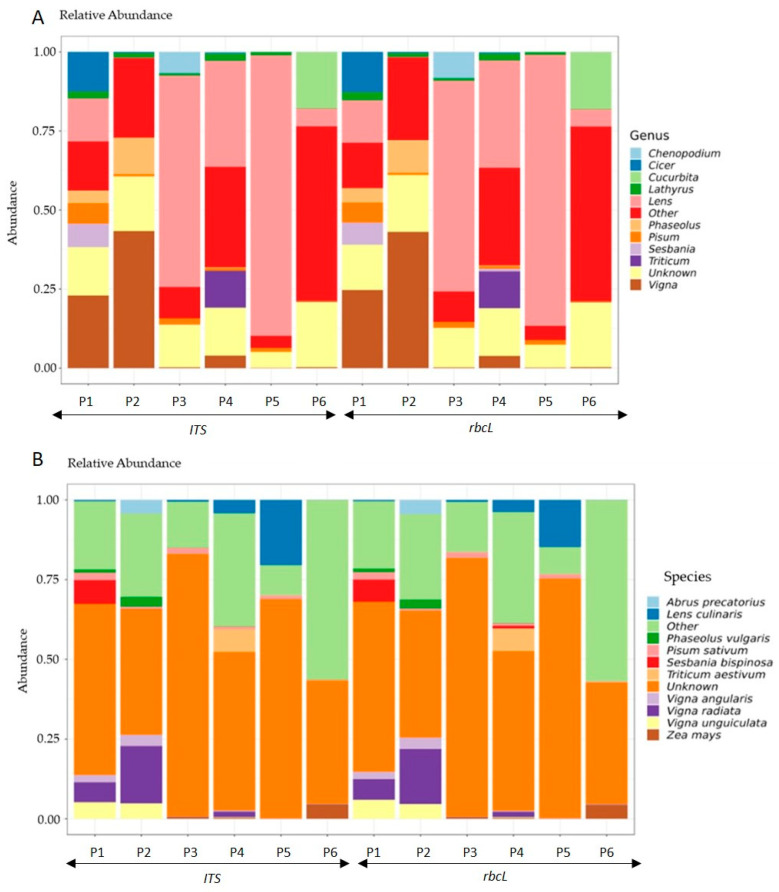
(**A**) Genera and (**B**) Species composition plot of all samples. The plot was generated by microbiome R package. The genera and species shown are the 11 most abundant. The remaining are included in the ‘Other’ category. The ‘Unknown’ is the abundance of reads whose genus is unknown because the taxonomy ID associated with them corresponds to a higher taxonomy group. The abundance in the *y*-axis represents the relative abundances in [0, 1] (microbiome R package).

Following the successful PCR amplification of the *ITS* and *rbcL* genes from the six selected products, the samples were subjected to sequencing analysis. The genera composition plot of all samples revealed a diverse taxonomic composition, with the 10 most abundant genera prominently displayed in Figure 1A. These included *Lens*, *Cicer*, *Phaseolus*, *Pisum*, *Triticum*, *Lathyrus*, *Vigna*, *Chenopodium*, and *Cucurbita*. Among the species identified, *Lens culinaris* (lentil), *Phaseolus vulgaris* (common bean), *Pisum sativum* (pea), *Triticum aestivum* (wheat), *Vigna angularis* (adzuki bean), *Vigna radiata* (mung bean), *Vigna unguiculata* (cowpea), and *Zea mays* (corn) were the most abundant (Figure 1B). The remaining genera and species in lower abundance were grouped into the ‘Other’ category, while the ‘Unknown’ category reflects reads whose taxonomy ID corresponds to a higher taxonomic group, making precise genus and species identification uncertain. Species discrimination in plants is more difficult than in animals due to greater levels of gene tree paraphyly, as well as a lesser amount of deposited data [43]. In animals, the most common marker for DNA barcoding is the *cytochrome c oxidase 1* (*CO1*) gene derived from the mitochondrial genome; however, in plants, a similar generic barcode is lacking due to its slow evolution and limited divergence [44,45,46]. Over the past years, several individual regions such as *matK*, *rbcL*, *trnH-psbA*, *ITS*, *trnL-F*, *5S-rRNA*, and *18S-rRNA* genes have been evaluated as individual candidates for their discriminatory properties in plant species identification [14]. A very common combination that has been broadly used are the *rbcL* and *ΜatK* regions as the *rbcL* gene is highly conserved, making it ideal for broad taxonomic identification. while *MatK*, can often provide extended variability [18,22,23,47,48]; however, it is underrepresented in GenBank libraries. Nonetheless, in closely related species, the discriminating ability of these two markers can be less informative [49,50]. Therefore, the addition of nuclear *ITS* (Internal Transcribed Spacers) region in combination to either the *matK* or the *rbcL* sequences, has been suggested as plant barcode in order to achieve maximum identification rates even in closely related species [14,15,51]. In the present study, the *rbcL* and *ITS* regions were selected to represent markers from both the chloroplast and nuclear genomes, ensuring that potential challenges in DNA extraction would not compromise the results. Based on data, shown in Figure 1, the *rbcL* and *ITS* markers were able to discriminate the genera’s contribution in biodiversity to a great extent; however, at the species level, a significant portion remained unresolved.

**Figure 2:**
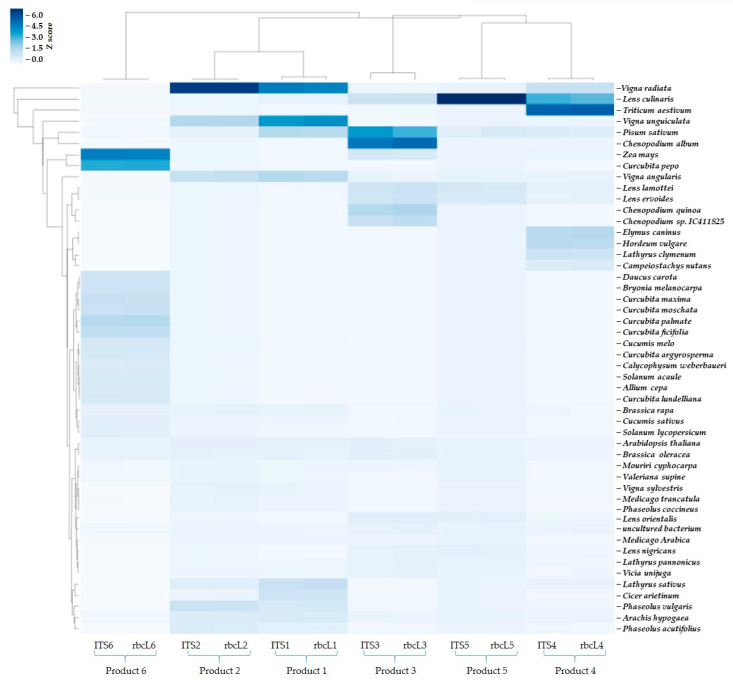
OTUs heat map with the 50 OTUs with the highest sum of read counts in all six samples for the two different genes sequenced. A higher Z-score (deep blue color) indicates a higher relative abundance or read count of a particular OTU in the sample compared to others.

**Figure 3:**
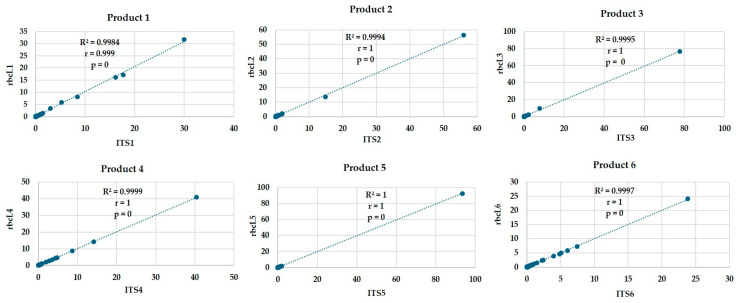
Correlation between *ITS* and *rbcL* genera read abundances across six plant-based food products. Scatterplots demonstrate the positive correlation between the *ITS* and *rbcL* relative read abundances for each genus per product. The correlations are shown in each graph with Pearson correlation coefficient and *p*-value.

The sequencing data were also used to demonstrate the highest read count operational taxonomic units (OTUs) and illustrate their distribution across the samples. The OTUs heat map revealed significant variation among the products, underscoring the diversity in plant species present across the samples. A total of 50 OTUs with the highest sum of read counts in all six samples for the two different genes sequenced are depicted in Figure 2. Among the OTUs shown, *Vigna radiata*, *Lens culinaris*, *Vigna unguiculata*, *Pisum sativum*, *Zea mays*, *Chenopodium quinoa*, several *Curcubita* species, *Phaseolus*, *Lathyrus clymenum*, and *Hordeum vulgare* demonstrated higher Z-score (intense blue color), indicating a higher relative abundance or read counts of a particular OTU in the sample compared to others. At the same time species such as *Daucus carota*, *Solanum lycopersicum*, *Allium cepa*, *Brassica rapa* and *B. oleracea*, and *Cicer arietinum* were detected in all products but with lower Z score. This highlights the sensitivity of the sequencing method, as it was able to detect and identify not only dominant species but also those present in traces such as *Arachis hypogea*, the common peanut, which is often present in small quantities in various products. The ability to sequence and analyze even species with low percentage in the samples demonstrates the sensitivity of the technique, making it an invaluable tool for comprehensive biodiversity assessments, provided that a GenBank library is available. This variation also validates the effectiveness of the *ITS* and *rbcL* genes for identifying the contribution of different plant taxa. It was also noted (Figure 2) that the OTUs for each gene were quite similar, demonstrating consistency in the taxonomic identification provided by these markers. This congruence further supports the reliability of the methods used in capturing and analyzing the biodiversity within the tested products. The consistency of the taxonomic composition identified by the two markers was further confirmed through Pearson correlation analysis, which yielded r values close to 1 and *p* values of 0, validating a strong and statistically significant correlation (Figure 3). This indicates that both markers produced highly similar read distributions, validating their combined use for reliable genus-level biodiversity assessment.

However, among the different species identified that were in accordance with the products ingredients, some unexpected species were also identified such as *Medicago truncatula*, *Sesbania bispinosa* or even some uncultured bacteria sequences. The detection of such species could be attributed to various factors such as potential contamination of the products during production or cross contact during processing but could also indicate the lack of reference sequences in reference databases. While some taxonomic groups are well represented in such databases, many others remain under-characterized, limiting accurate identification [16]. Furthermore, DNA barcoding of herbal or multi-species food products presents particular challenges, often due to variable PCR efficiency caused by degraded DNA, differing gene copy numbers, or PCR amplification bias [43]. The identification of the species *Abrus precatorius* and *Sesbania bispinosa* in the present study, both members of the *Fabaceae* family, like many legumes, could illustrate some of the DNA barcoding limitations, at least with the markers and/or the taxonomic family used. While *Sesbania bispinosa* is a widespread weed, *Abrus precatorius* is a herbaceaous flowering plant known for its toxic seeds. Therefore, it is highly likely that the identification of this species is misleading, as it does not probably represent a true ingredient. Similar findings need to be further analyzed using additional DNA barcoding markers or alternative combinations that will validate their accuracy and rule out potential false positives.

### 3.3. Comparison of Label Composition and Sequencing-Based Genera Identification

**Figure 4:**
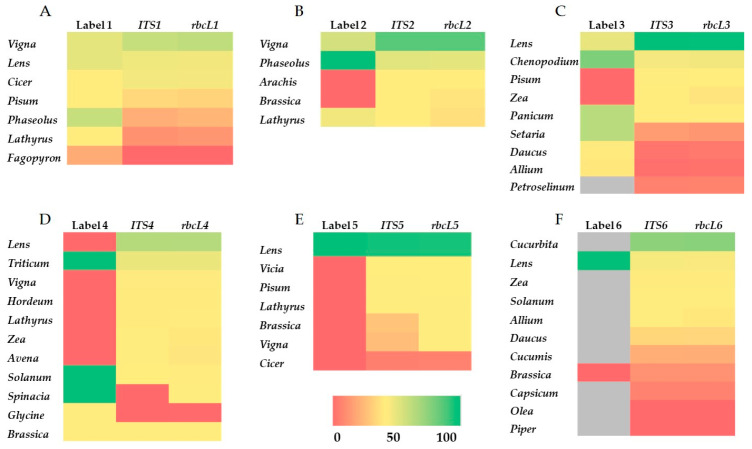
Comparison of label composition and *ITS*, *rbcL*-based genus detection across six plant-based food products. Heat maps show the correlation between the labeled weight percentage (% *w*/*w*, column 1) of each ingredient and its corresponding *ITS* and *rbcL* read abundance (columns 2 and 3). Panels (**A**–**F**) correspond to Products 1–6, respectively. Color scale ranges from red to green, with green, yellow and red representing no, intermediate and strong match between read abundance and weight percentage, respectively. Gray color represents lack of labeled weight percentage.

Beyond biodiversity assessment, DNA barcoding can serve as a powerful tool in food traceability and authentication, providing reliable identification of species. To evaluate the accuracy and limitations of DNA barcoding in reflecting actual ingredient composition, we compared the declared label content of all six products with the taxonomic profiles obtained through *ITS* and *rbcL*-based sequencing. For each product, a heat map was generated to compare the declared ingredients percentage by weight (% *w*/*w*) in each label with the corresponding percentage of the *ITS* and *rbcL* read abundances (Figure 4). Only species listed on the product label or species showing high abundance were included in the heat map, allowing us to focus specifically on the accuracy of ingredient representation and to detect any potential mislabeling. The molecular data strongly supported the ingredient composition with minor exceptions. More specifically, in Product 1 the genera *Phaseolus*, *Lens*, *Cicer*, *Pisum* and *Lathyrus* were identified, while genus *Fagopyron* which was also listed on the label, was not detected (Figure 4A). In Products 2 and 3, all labeled genera were identified. Additionally, low levels of *Arachis* and *Brassica* were detected in Product 2 while traces of *Pisum* and *Zea* were found in Product 3 although not declared on the label (Figure 4B,C). In Product 6 all labeled ingredients were molecularly identified, even those in traces (Figure 4F). In contrast, Product 5, which listed genus *Lens* as the only ingredient, showed presence for several other genera such as *Vicia*, *Pisum*, *Brassica*, *Lathyrus*, *Vigna* and *Cicer*, suggesting possible cross-contamination or mislabeling (Figure 4E). Finally, in Product 4 (pasta made from wheat with tomato and spinach), although all genera were identified through *ITS* and *rbcL* sequencing, the main expected genera, *Triticum* was underrepresented in the sequence data (% abundance) compared to the percentage of the label. Instead, other genera such as *Lens*, *Vigna*, *Hordeum*, *Lathyrus*, *Zea* and *Avena* were identified, indicating potential substitution of wheat flour with alternative cereal or legume flours (Figure 4D). These results provide strong evidence for the application of DNA barcoding and especially *ITS* and *rbcL* markers not only in biodiversity assessment but also in food authentication, enabling the detection of mislabeled products and fraudulent substitutions [18,44,52]. In the food supply sector, the misrepresentation of species origin is a common problem. DNA barcoding enables the identification of food products at a molecular level, verifying whether the genera and species listed on the label match the actual ingredient contents [53,54]. During the last years, numerous studies have been conducted to address the biodiversity, authentication, and mislabeling of plant-based medicinal and food products [47]. Standard barcode regions, including *ITS* and *rbcL* markers used in the present study, have been employed to discriminate herbal and medicinal plant-based products, spices, herbal infusions, salad and seeds mixtures, as well as to authenticate the Designation of Origin of plant products as saffron or to detect adulteration in plant products [55,56,57,58,59,60,61]. However, in some cases, the complexity of the mixtures or the excessive food processing could affect the ability of DNA barcoding to recover intact sequences for analysis [41]. These observations suggest that sequencing read abundance is not directly proportional to ingredient weight, even under controlled sample preparation conditions. For example, in the case of plants, successful PCR of barcoding regions is often inhibited by the presence of secondary metabolites. However, modifications in extraction methods, primer sequences, and the use of an enhanced DNA polymerase can usually overcome such problems. The combining of barcodes from multiple loci has also been used successfully [14]. Collectively, these findings demonstrate that the complementary use of *ITS* and *rbcL* markers enables reliable genus-level identification across a wide range of plant-based commercial products, while *ITS* contributes valuable species-level resolution. By applying *ITS* and *rbcL* DNA barcoding to a range of commercially available plant-based products with different processing levels, we were able to assess the consistency between the two markers, evaluate the impact of processing on DNA identification, and compare results with product labels. These findings provide practical insights into using DNA barcoding for biodiversity assessment and food authentication in complex commercial products.

## 4. Conclusions

This study highlights the use of DNA barcoding as a robust and sensitive method for revealing plant-based product biodiversity and ensuring ingredient authenticity. The use of *rbcL* and *ITS* markers successfully profiled the plant composition of six plant-based commercial products that have undergone different levels of processing. Major genera such as *Lens*, *Cicer*, *Lathyrus*, *Chenopodium*, *Phaseolus*, *Pisum*, *Vigna*, and *Triticum* were dominant; however, genera and species of lower abundance were also identified, significantly contributing to product biodiversity. The approach proved sensitive enough to detect both major and trace species, providing a detailed snapshot of taxonomic diversity. However, limitations at the species level were evident, with some taxa remaining unidentified or misassigned, pointing to the need for additional markers or the deposition of more taxa sequences in GenBank libraries as references. The strong correlation between *ITS* and *rbcL* sequencing data was indicated by OTUs distribution and Pearson correlation, supporting their combined use for reliable genus-level biodiversity assessment. Heat map analyses confirmed high concordance between label claims and sequencing results in most cases, though undeclared species and absent labeled taxa were also observed, highlighting potential mislabeling or cross-contamination. Overall, this proof-of-concept study confirms that DNA barcoding can be a valuable tool, not only for food traceability and authentication but also for the in-depth characterization of plant biodiversity in complex food matrices, taking into account the ongoing accumulation of molecular reference data. The focus was on providing the first step toward uncovering and visualizing the biodiversity present in mixed crop food products. By making this diversity visible, our work provides baseline information that can later be translated into more complex BIOVALUE metrics, combining biodiversity with nutritional and functional values. In this context, future perspectives include expanding this approach to a broader range of commercial products, integrating additional DNA barcoding markers to improve species-level resolution, developing dedicated reference databases for food biodiversity, and combining biodiversity data with nutritional and functional information to better assess the overall value of food products.

## Figures and Tables

**Table 1:** Selection of final products tested for DNA extraction efficiency. The array includes commodities with varying ingredient compositions and processing methods, such as different salt and sugar contents, additives, and thermal processing techniques. Products 1 to 3 were dried mixtures of legumes and seeds, products 4 and 5 were types of pasta, made with wheat, lentils, herbs, and vegetables. Product 6 was a frozen blend of legumes and vegetables. Products 7, 8, and 10 were tomato-based items processed to different extents, and product 9 was a canned mix of legumes and vegetables. Each product represents a distinct category, ensuring a comprehensive assessment of the DNA extraction process.

Samples	Ingredients	Thermal Processing or Additives
Product 1	10% chickpeas, 10% lathyrus seeds, 10% Beluga lentils, 10% red lentils, 10% red beans, 10% black-eyed beans, 10% white beans, 10% green beans, 10% crashed peas, 5% fagopyrum seeds, 5% black beans, 5% borlotti beans	no
Product 2	40% white beans, 30% borlotti beans, 10% lathyrus seeds, 10% black-eyed beans, 10% mung bean (*Vigna radiata*)	no
Product 3	38% white quinoa, 31% millet, 13% red lentils, 13% red quinoa, 4% dehydrated carots, 0.5% dehydrated leeks, Dehydrated parsley	no
Product 4	Durum wheat semolina, 4% dehydrated tomatoes, 3% dehydrated spinach, traces of soya and mustard	yes
Product 5	Red lentils flour	yes
Product 6	36.5% lentils, red pepper, corn, onion, carrots, zucchini, tomato, extra virgin oil, salt and black pepper	yes
Product 7	28–30% tomato juice solids, salt	yes
Product 8	60% peeled plum tomatoes, 40% slightly concentrated tomato juice, acidity regulator, citric acid	yes
Product 9	beans, green beans, carrots, white turnip, salt	yes
Product 10	sun dried tomatoes, sulfur dioxide, salt	yes

## Data Availability

The original contributions presented in this study are included in the article/Supplementary Material. Further inquiries can be directed to the corresponding authors.

## Supplementary Materials

The following supporting information can be downloaded at: https://www.mdpi.com/article/10.3390/foods14183256/s1, Figure S1: PCR amplification of the ~800 bp of the *ITS* gene fragment in plant-based food products. Each lane corresponds to the amplification of the gene in products 1–10, respectively, with the last lane representing the negative control. No amplification was observed in Products 7–10 indicating the inhibition of PCR in these samples. Table S1: DNA quality and yield assessment using different DNA extraction methods.
