# Supplementary material for: DNA Barcoding for Tracing Biodiversity in Mixed Crop Food Products: A Proof of Concept Within the BioValue Project
Source: Foods. 2025 Sep 19;14(18):3256. doi: 10.3390/foods14183256 (PMC12469653; doi:10.3390/foods14183256)
Supplement: Supplementary file 1 [file foods-14-03256-s001.zip › foods-3859245-supplementary.pdf]

Table S1. DNA quality and yield assessment using different DNA extraction methods.

	Silica Column-Based Method			C-TAB-Based Method		
**Title 1**	**DNA Quantity (ng/µL)**	**Absorbance 260/280**	**Absorbance 260/230**	**DNA Quantity (ng/µL)**	**Absorbance 260/280**	**Absorbance 260/230**
Product 1	91.76	1.6	0.68	884.84	1.99	1.29
Product 2	39.71	1.74	0.95	59.82	1.9	1.24
Product 3	3.48	1.99	−0.7	1011.27	2.09	1.99
Product 4	3.27	1.64	2.91	518.95	2.02	1.77
Product 5	1.65	2.92	−0.38	70.76	0.716	1.98
Product 6	7.46	1.53	0.93	382.78	1.95	1.55
Product 7	41.07	1.8	0.58	396.46	1.48	1.16
Product 8	8.46	1.32	0.41	93.27	1.78	1.09
Product 9	5.06	3.78	0.97	482.66	2.02	1.51
Product 10	94.51	1.69	0.92	529.86	1.51	1.2

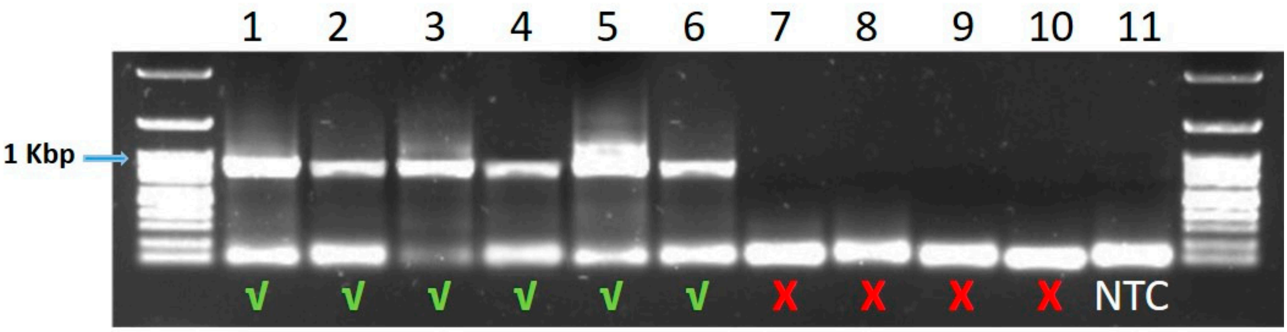

Figure S1. PCR amplification of the ~800 bp of the *ITS* gene fragment in plant-based food products. Each lane corresponds to the amplification of the gene in products 1–10, respectively, with the last lane representing the negative control. No amplification was observed in products 7–10, indicating the inhibition of PCR in these samples.
